# Mixed Metals Toxicity: More than the Sum of Its Parts?

**DOI:** 10.1289/ehp.120-a35b

**Published:** 2012-01-01

**Authors:** Angela Spivey

**Affiliations:** Angela Spivey writes from North Carolina about medicine, environmental health, and personal finance.

Most toxicologic studies focus on one chemical or toxicant at a time, although in the real world people are exposed to multiple substances at once. Metals, for example, are commonly found in combinations in air, water, and food. Now researchers have conducted a prospective epidemiologic study to examine neurodevelopmental effects of manganese–lead coexposure [*EHP* 120(1):126–131; Claus Henn et al.].

The study included 455 children enrolled at birth in a Mexico City longitudinal cohort study, who were followed until 36 months of age. The researchers measured blood levels of lead and manganese at 12 and 24 months of age. Cognitive, motor, and language development were assessed every 6 months from 12 to 36 months of age using the Bayley Scales of Infant Development–II. Both lead and manganese exert their toxic effects on child development primarily through the central nervous system.

Exposure to manganese and lead simultaneously was associated with greater deficits in both mental and psychomotor development than expected based on the estimated effects of exposure to either metal alone; this is consistent with a synergistic effect on neurodevelopment. Associations were greatest at 12 months, where Mental Development Index scores were 2.16 points lower per 1-unit increase in blood lead among children with high manganese compared with children with midrange manganese. Psychomotor Development Index scores at 12 months were 0.97 points lower per 1-unit increase in blood lead among children with high manganese compared with children with midrange manganese. The authors suggest that the developmental period up to 12 months may be particularly sensitive to this interaction.

The differences in mental and psychomotor development scores were small for individuals, but the authors write that these differences may have population-level consequences if they cause the entire distribution of neurodevelopment scores to shift. This would increase the number of children eligible for diagnosis with neurodevelopmental problems (who would then require treatment) as well as decrease the average IQ.

The study’s strengths include a stable study population with little attrition as well as the repeated measurements of neurodevelopment between 12 and 36 months, which allowed the researchers to evaluate associations over time and offered greater statistical power than cross-sectional analyses. Weaknesses of the study included the lack of formal exposure assessment, which means the sources of metal exposure were unknown. In addition, the neurodevelopment test instruments used in the study were not normalized to the Mexican population, so the resulting scores are relatively low compared with the expected mean score of 100 in a U.S. population and cannot be generalized to other populations.

